# Parenteral Anticoagulation at First Medical Contact Improves Infarct Related Artery Patency in STEMI

**DOI:** 10.3390/jcm13061710

**Published:** 2024-03-16

**Authors:** Vlad Bataila, Nicoleta-Monica Popa-Fotea, Cosmin Cojocaru, Lucian Calmac, Cosmin Mihai, Marian-Bogdan Dragoescu, Vlad Ploscaru, Mugur Marinescu, Vasile Iliese, Anamaria-Georgiana Avram, Raluca-Elena Mitran, Radu-Gabriel Vatasescu

**Affiliations:** 1 Department of Cardiology, Clinical Emergency Hospital of Bucharest, Calea Floreasca 8, 014461 Bucharest, Romania; vladbataila@yahoo.co.uk (V.B.); fotea.nicoleta@yahoo.com (N.-M.P.-F.); cojocaru.r.b.cosmin@gmail.com (C.C.); lcalmac@gmail.com (L.C.); cosmihalp@yahoo.com (C.M.); dr.marianbogdan@gmail.com (M.-B.D.); vlad_ploscaru86@yahoo.com (V.P.); mugurmarinescu@gmail.com (M.M.); ralucamitran972@gmail.com (R.-E.M.); 2 Department of Cardiology, Carol Davila University of Medicine and Pharmacy, Eroii Sanitari Bvd. 8, 050474 Bucharest, Romania; 3 Laboratory of Interventional Cardiology, Carol Davila Central Military Universitary Emergency Hospital, Mircea Vulcanescu Str. 88, 010825 Bucharest, Romania; iliesevasile@yahoo.com; 4 Department of Cardiology, Bagdasar-Arseni Emergency Clinical Hospital, Soseaua Berceni 12, 041915 Bucharest, Romania; anamaria.g.avram@gmail.com

**Keywords:** ST-elevation myocardial infarction, percutaneous coronary intervention, heparin, infarct-related artery patency, bleeding rates, reperfusion

## Abstract

(1) **Background**: Acute ST-segment elevation myocardial infarction (STEMI) remains one of the main morbidity and mortality contributors worldwide. Its main treatment, primary percutaneous coronary intervention (pPCI), can only be performed with a high anticoagulation regimen, usually with heparin. There is still not enough evidence regarding the timing of heparin administration. (2) **Methods**: We conducted a multicenter observational study of 614 consecutive STEMI patients treated between 2017 and 2019. We split the population in two groups: one that received heparin at the first medical contact, as early as possible, and the second group that received heparin at the PCI capable center or in the cath lab. (3) **Results**: There was a significantly higher rate of infarct-related artery (IRA) patency at the time of the coronary angiogram in the pre-transfer heparin group than in the on-site heparin group, 44.7% vs. 37.3%, *p* = 0.042. Also, the early heparin group received shorter and wider stents. There was no difference in bleeding rates or in the in-hospital and two-year mortality rates. (4) **Conclusions**: Early administration of heparin leads to a higher rate of reperfusion in the IRA, before pPCI, with significant related benefits, such as better stent implantation parameters, without increased bleeding rates.

## 1. Introduction

Ischemic heart disease (IHD) is the dominant cause of mortality in Romania [1]. Acute ST-segment elevation myocardial infarction (STEMI) is the most severe type of IHD and early treatment is critical for improving outcomes. The most frequent mechanism of STEMI is thrombosis on an unstable atherosclerotic plaque [2]. The cornerstone of STEMI management is primary percutaneous coronary intervention (pPCI), which mandates concomitant antithrombotic treatment. The guideline-directed standard of care of parenteral anticoagulation in STEMI is heparin administered at the time of diagnosis, as class I indication, evidence level A, because of its favorable risk/benefit profile [2]. However, high-quality evidence establishing the benefits of heparin administration at an earlier point in STEMI patients undergoing primary PCI is lacking [2]. Therefore, the effect of early heparin administration at first medical contact (FMC) on short and long-term outcomes is still being assessed [3,4]. Heparin activates antithrombin III, which inactivates thrombin and factor Xa, which blocks the conversion of fibrinogen to fibrin, thus inhibiting clot formation [5]. As a result, the main advantage of early heparin administration is the potential to increase the rate of coronary artery revascularization, improving the infarct-related artery patency. However, one disadvantage of heparin use is the risk of bleeding. The incidence of major bleeding events due to heparin administration in patients with acute coronary syndrome is 4.5% (386/8606 patients), according to a study by Petersen et al. [6]. Another side effect of heparin use is heparin-induced thrombocytopenia (HIT), which can be categorized into two types [7]. Type 1 HIT is a non-immune mediated reaction, characterized by a mild and transient thrombocytopenia, usually asymptomatic [7]. Type 2 HIT is an immune-mediated reaction that causes a hypercoagulable state due to platelet activation, leading to increased risk of thrombosis and potentially life-threatening thrombotic complications [7]. This study aims to evaluate the effect of FMC heparin on infarct-related artery (IRA) patency and bleeding events in STEMI patients in Romania.

## 2. Materials and Methods

### 2.1. Patient Selection 

We conducted an observational, retrospective analysis of all consecutive STEMI patients transferred from non-PCI centers to pPCI capable centers from October 2017 to February 2019. The diagnosis of STEMI was based on European guidelines and the Fourth Universal Definition of Myocardial Infarction standard criteria [2,8]. The study was approved by The Ethical Committee of Emergency Clinical Hospital Bucharest, Romania, no 370/03.07.2017.

Patients treated by thrombolysis or patients directly taken by the ambulance service to pPCI centers were excluded. The population was divided into two different subgroups: Group A, treated with heparin at FMC, as soon as possible, under 30 min (pre-transfer group) and Group B, who exclusively received heparin at the pPCI center (the post-transfer group). Heparin was administered in guideline-directed doses. There was no enoxaparin administered in this study. Periprocedurally, the interventional cardiologists freely administered additional heparin according to internal protocol and experience, frequently guided by an activated clotting time (ACT) of over 250 s. Also, glycoprotein IIb/IIIa inhibitors, thrombus aspiration, direct stenting and the type of stents were determined by the interventional cardiologist. 

### 2.2. Endpoints

The study end-points were:

- Rate of infarct-related artery (IRA) patency on coronary angiogram;
- Rate of in-hospital and post-discharge two-year mortality rates;
- Rate of in-hospital bleeding events from initial symptoms to discharge prior to the moment of discharge: major bleeding (Bleeding Academic Research Consortium (BARC) score > 2) any bleeding, access site-related bleeding;
- Stroke rate from initial symptoms prior to the moment of discharge.

### 2.3. Statistical Analysis

Statistical analysis was performed using SPSS version 26 (IBM Corp., Armonk, NY, USA) software. Continuous data were expressed as mean ± standard deviation (SD) for normally distributed data and median (IQR) for non-normally distributed data. Categorical data were expressed as percentage (count). The normality of data was evaluated by the Kolmogorov–Smirnov test. Categorical variables were compared using the Fisher’s exact test/chi-square analysis and continuous variables were compared using Student’s *t*-test if normally distributed and non-parametric tests (Mann–Whitney U Test) if not. 

## 3. Results

**Figure 1:**
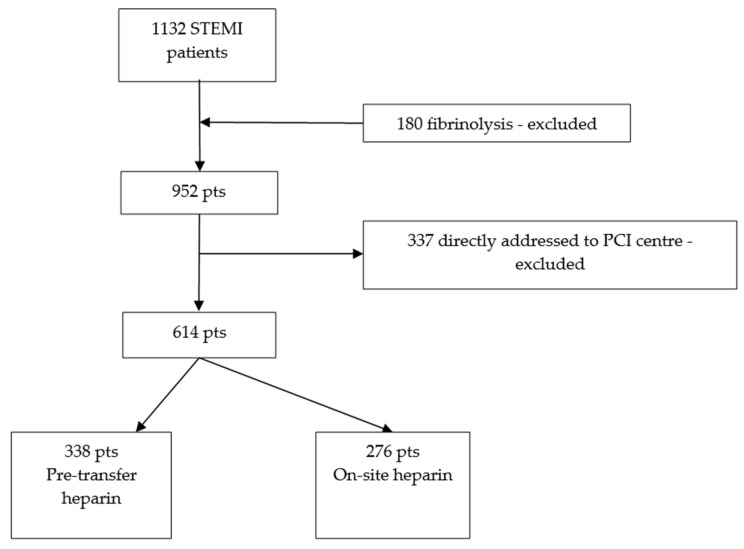
Study population selection design. STEMI, ST-elevation myocardial infarction; PCI, percutaneous coronary intervention; pts, patients.

Six hundred and fourteen (*n* = 614) patients were included in this analysis after exclusion of 180 patients that were treated by fibrinolysis and 338 patients with FMC location addressed directly to pPCI centers (Figure 1). The patients treated by fibrinolysis were excluded from the study since it is not possible to determine whether the effects on IRA patency, as well as bleeding rates, were due to the fibrinolytic agent or heparin administration. The patients that presented directly to pPCI centers were also excluded because they had a very short FMC-to-balloon time and a very short action time of the antithrombotic medication; therefore, the effect of heparin administration on these patients cannot be assessed properly. All transferred patients were included in the study. Although door-to-balloon time is considered more important, we assessed FMC-to-balloon time, since only transferred patients were included in our study. 

The baseline characteristics of the two subgroups are shown in Table 1. The mean FMC-to-balloon time was 206.6 ± 256.3 min in Group A and 187.7 ± 165.0 in group B. Although the desirable FMC-to-balloon time is below 120 min, since only transferred patients are included in this study, the FMC-to-balloon time in our study is in line with the current international literature on this subject [9]. All patients in both groups received aspirin at FMC, so the ratio of non-aspirin use is zero. There were no significant baseline differences between the two study groups, except for the P2Y12 inhibitor administration (either clopidogrel or ticagrelor) which was more frequent in the early heparin group 97% vs. 80.1%, *p* < 0.001. 

Table 2 summarizes characteristics regarding treatment results and complications in Group A versus Group B. There was a significantly higher rate of IRA patency in the pre-transfer heparin group than in the on-site heparin group, 44.7% vs. 37.3%, *p* = 0.042. There were no significant differences regarding major bleedings, any bleeding or minimum hemoglobin. There were fewer access site complications in the early heparin group than in the on-site heparin group, although this did not reach statistical significance (0.9 vs. 2.9, *p* = 0.06). There were no differences regarding in-hospital or post-discharge two-year mortality between the two groups. Stent diameter and stent length were higher (3.15 vs. 3.06, *p* = 0.037) and shorter in Group A versus Group B, respectively (22.4 vs. 26.6, *p* = 0.10).

The multivariate analysis performed (Table 3) found that the independent predictors for a patent IRA were early administration of heparin (OR 1.50, CI 95% 1.06–2.13, *p* = 0.019), anterior MI (OR 1.45, CI 95% 1.04–2.03, *p* = 0.026) and diabetes (OR 1.59, CI 95% 1.10–2.28, *p* = 0.012). Notably, after multivariate adjustment, early administration of P2Y12 inhibitors did not contribute significantly.

## 4. Discussion

### 4.1. Effect of Pre-Transfer Heparin Effect on IRA Patency 

Patients with STEMI require parenteral anticoagulation, usually with heparin, in order to help achieve reperfusion and also to minimize thrombotic adverse events during primary PCI. However, the present guidelines do not mention whether the anticoagulation therapy must be administered before (at first medical contact) or during primary PCI [2]. The rationale for earliest possible administration of heparin is to permit endogenous fibrinolysis to take action and decrease the thrombotic burden of the culprit lesion in the infarct-related artery and the total thrombus formation, to allow for spontaneous reperfusion. On one hand, this is of utmost benefit because it is well-known that increased thrombus burden in the culprit lesion is strongly associated with adverse outcomes [10,11,12]. On the other hand, there is evidence that there are some individuals who have an impaired endogenous fibrinolysis that could benefit even more from the prolonged action of heparin [13]. 

There are several studies that deal with the moment of heparin administration. The largest study was the Swedish Coronary Angiography and Angioplasty Registry (SCAAR), which comprised 41,631 patients. In a propensity score-matched analysis, it showed a rate of coronary occlusion of 62% in the early heparin group versus 71% in the no heparin group, with the number needed to treat being 12 without an increase major bleeding events [3]. In addition to improving IRA patency at the time of PCI, the authors inferred a potential reduction in 30-day mortality in radial artery access patients. However, this effect was not robust across all sensitivity analyses and should be further evaluated. Although an extremely large population, the two study arms have markedly different population characteristics, so it is possible that there is some residual confounding. Furthermore, compared with SCAAR, which does not include relevant information including time-to-balloon time or time-to-FMC, or this information is not further analyzed, our study includes this information. Differently from our study, SCCAR shows a benefit for 30-days mortality. One factor that may contribute to this disparity is the time-to-FMC and time-to-balloon, but we do not have data to compare, as this information is not included in the SCAAR research. 

Another study [14] showed a striking reduction in short- and long-term mortality which was independently predicted by pre-transfer heparin, alongside age, radial access, and cardiogenic shock. Importantly, ischemia times in the pre-transfer subgroup were half of those observed in the post-transfer subgroup. In our study, there was no difference between the time-to-FMC or the time-to-balloon, that may explain the lack of benefit on survival. Compared with the mentioned above study, we lack information about the incidence of cardiogenic shock, a major impacting factor on 30-day mortality. 

Giralt et al. showed that early heparin administration results in higher artery patency in a time-dependent manner and improves clinical outcomes [15]. Also, they showed that the sooner the heparin is administered, the better, with improved clinical outcomes [15]. An insight from the TOTAL clinical trial, using the same population but analyzing the effect of upstream anticoagulation with heparin on artery patency and thrombus, showed that pre-PCI anticoagulation was associated with improved flow and reduced thrombus burden, with non-significant differences regarding clinical outcomes [16]. Also, another post-hoc analysis, of the TASTE trial, showed that heparin pre-treatment was associated with lower risk of intracoronary thrombus and vessel occlusion with no difference on hard outcomes [17]. 

Regarding antiplatelet pre-treatment, our analysis showed that despite a higher number of patients receiving any kind of dual antiplatelet therapy (DAPT) at FMC, after adjusting for a number of predictors, DAPT pre-treatment did not influence IRA patency. This is in line with the DAPT pre-treatment keystone study, ATLANTIC [18] and also with the new ESC Acute coronary syndrome guidelines [2].

In our study, one of the main characteristics that must be discussed is the long ischemia time, including the time from FMC to balloon. The approximate 200 min average time from the heparin dose to pPCI is in line with what Giralt [15] reported, but the symptom onset to FMC time is much higher in our population (290 min vs. 77 min). The smaller, although still significant, difference compared to ones reported in other similar clinical trials regarding artery patency could be explained by the long ischemia times.

Having IRA patency at the beginning of the PCI procedure was shown to lead to markedly better outcomes, and this is probably the most important benefit of early pre-PCI heparin administration [19,20]. Although pre-treatment with heparin has produced mixed clinical outcomes in similar studies including our own, one must not ignore several other benefits that come with a patent infarct-related artery, that cannot be as easily quantified [19,21]. Foremost, the pPCI procedure takes place at a different pace, with less patient discomfort, more time to correctly evaluate the target lesion and to proceed with the optimal strategy and also less stress on the revascularization team. 

### 4.2. Effect of Pre-Transfer Heparin Administration on Stent Diameter and Length

Regarding the procedural impact of heparin pre-treatment, our study showed that patients pre-treated with heparin received significantly shorter and larger stents. We do not think this is an incidental finding, although it was not reported before, because it is known that culprit lesions from infarct-related arteries that have higher thrombus burden have poorer visibility of the real lesion [22]. Also, there is a well described relationship between thrombus and arterial spasm [23]. These, together with other well-known procedural pitfalls in pPCI (i.e., impaired nitroglycerine administration, short duration, difficult lesion preparation, intention of avoiding post-dilatation) contribute to the adverse procedural outcomes in acute STEMI. Total stent length and diameter are one of the main procedural predictors of PCI late adverse events [24,25,26]. Although the two-year mortality rate was not significantly different between Group A and Group B, we do not have data regarding repeated target lesion revascularization or longer follow-up. 

### 4.3. Risk of Pre-Transfer Heparin Administration Regarding Bleeding Events

Pre-treatment with heparin was shown to be safe in our study as well as in all of the similar trials [8,15,16,17], with no increase in bleeding events, including major bleeding, stroke or vascular access bleeding. That is partly because heparin is a well known and familiar drug, but also because heparin pre-dose leads to an increase of duration of anticoagulation only as long as the time between the FMC to balloon. However, detailed subgroup analyses in the SCAAR registry [3] show significantly higher rates of major bleeding events in those with non-radial access, those who weigh less than 60 kg and those older than 75 y.o., who are generally regarded as frail patients, prone to more frequent procedural complications.

## 5. Limitations

1. Retrospective analysis with a relatively smaller sample compared to previously published registry analyses.
2. Lack of routine evaluation of pre-angiography anticoagulation status which may impact both IRA patency and bleeding risk and is influenced by:
  - Variable pre-transfer heparin dosing (5000 UI vs. weight-adjusted dose);
  - Long time intervals from FMC (i.e., pre-transfer heparin administration) which may diminish residual anticoagulant effect at the time of coronary angiography.
3. Confounding factors regarding antithrombotic therapy—antiplatelet therapy loading or omission of loading at FMC, type of antiplatelet therapy, simultaneous opioid administration.

## 6. Conclusions

The data consistently show that early administration of heparin leads to an increased rate of coronary artery patency at the moment of primary PCI in STEMI patients. Moreover, early administration of heparin also leads to some procedural benefits such as the use of larger and shorter stents. Across all data, early heparin administration turned out to be safe in our study, with very low bleeding rates. 

## Figures and Tables

**Table 1:** Baseline characteristics.

	Group A (Early Heparin) (*n* = 338)	Group B (Heparin On Site) (*n* = 276)	*p*
Age, years	62.3 ± 12.9	63.1 ± 14.0	0.45
Female sex, *n*%	91 (26.9%)	79 (28.6%)	0.64
Smoking, *n*%	205 (62.1%)	175 (65.1%)	0.45
Hypertension, *n* %	252 (74.6%)	208 (75.4%)	0.71
Dyslipidemia, *n* %	184 (54.5%)	154 (55.8%)	0.54
Diabetes mellitus, *n* %	77 (23.3%)	39 (29.2%)	0.10
Previous MI, *n*%	25 (7.4%)	22 (8.0%)	0.79
Previous angina, *n* %	176 (52.1%)	139 (50.4%)	0.67
Previous PCI, *n*%	15 (4.5%)	17 (6.2%)	0.09
Previous stroke, *n* %	27 (8%)	26 (9.4%)	0.53
Cancer, *n* %	13 (3.9%)	21 (7.6%)	0.15
Anterior MI, *n* %	144 (42.6%)	114 (41.3%)	0.74
Radial procedure, *n* %	293 (86.7%)	237 (85.9%)	0.39
Manual thrombectomy, *n* %	107 (31.7%)	87 (31.5%)	0.97
Aspirin on FMC or pretreatment, *n* %	338 (100%)	276 (100%)	0.73
DAPT (Aspirin + P2Y12 inhibitors) on FMC, *n* %	328 (97.0%)	221 (80.1%)	<0.001
Time from symptoms to FMC, minutes	290.6 ± 310.1	341.1 ± 460.2	0.31
Time FMC to balloon, minutes	206.6 ± 256.3	187.7 ± 165.0	0.30

**Table 2:** Results.

	Group A (Pre-Transfer) (*n* = 338)	Group B (Heparin on-Site) (*n* = 276)	*p*
Patent infarct related artery, *n* %	151 (44.7%)	103 (37.3%)	0.04
Major bleeding (requiring transfusion), *n* %	1 (0.3%)	0	0.45
Any bleeding, *n* %	11 (3.2%)	7 (2.0%)	0.70
Minimum hemoglobin, g/dl	12.8 ± 2.2	12.9 ± 2.3	0.63
Vascular access complications, *n* %	3 (0.9%)	8 (2.9%)	0.06
Periprocedural stroke, *n* (%)	0 (%)	1 (0.4%)	0.26
pPCI during index procedure, *n* (%)	295 (87.3%)	252 (91.3%)	0.12
LAD, *n* (%)	152 (45%)	127 (45.5%)	0.36
LCX, *n* (%)	49 (14.5%)	47 (17%)	
LM, *n* (%)	6 (1.8%)	1 (0.4%)	
RCA, *n* (%)	131 (38.8%)	101 (36.6%)	
Stent diameter, mm	3.15 ± 0.66	3.06 ± 0.76	0.03
Stent length, mm	22.4 ± 11.2	26.6 ± 10.1	0.04
In-hospital mortality, *n* (%)	31 (9.2%)	30 (10.9%)	0.48
Two-year total mortality, *n* %	58 (17.1%)	54 (19.5%)	0.40

**Table 3:** Multivariate analysis.

Variable	Odds Ratio	CI 95%	*p*
Age, years	1.00	0.99–1.01	0.507
Gender, male	1.17	0.79–1.72	0.412
Smoking	1.23	0.86–1.76	0.256
Hypertension	0.81	0.54–1.22	0.329
Diabetes mellitus	1.59	1.10–2.28	0.012
Anterior STEMI	1.45	1.04–2.03	0.026
Early P2Y2 inhibitor administration	0.67	0.38–1.16	0.160
Heparin administration at FMC	1.50	1.06–2.13	0.019

## Data Availability

At request from the corresponding author.
